# Indents

**Published:** 1908-04-15

**Authors:** 


### INDENTS.

☞Brief Articles of Techinical or Scientific Value will receive notice and comment. Contributions invited.

**Ransom & Randolph Company Reorganization.** A reorganization of the Ransom & Randolph Co. has been effected on account of the deaths last December of the company's founders, Messrs. Ransom and Randolph, and the business turned over to a new company which was incorporated at Columbus, February 13, with a capital stock of $150,000. C. S. Bigelow has been elected president, with F. C. Crandell, secretary-treasurer.

**Institute of Dental Pedagogy.** At the recent meeting in New Orleans the institute elected the following officers for the ensuing year: President, Dr. W. E. Wilmott, of Toronto, Ontario; vice-president, Dr. E. Hillyer, of Brooklyn, N. Y.; secretary-treasurer, Dr. B. E. Lischer, of St. Louis; executive board, Dr. D. M. Gallie, of Chicago; Dr. J. Q. Byram, of Indianapolis, Ind.; Dr. H. E. Frissell, of Pittsburg, Pa. The next meeting will be held in St. Louis.

**A Dentist's Earning Capacity.** A dentists' earning capacity, if a successful and progressive practitioner, should increase as he gains experience and expertness. This does not come from larger fees alone. Ability to do more in a given time; being able to decide quickly what to do, and how to do it; less time wasted in unprofitable experiments, and so managing one's patients as to be kept fully employed during office hours, are far more important factors in increasing one's earning capacity than increased fees.—Dr. Chas. F. Bonsall in *Brief*.

**Antiseptic Spray for Teeth and Gums.** In cleaning teeth I frequently follow the stick and ribbon floss with the brush wheel used upon the coronal surfaces. After this is all done, I use an antiseptic spray under a pressure of thirty pounds, and go all over the teeth, between them, and especially under the gum margin, to thoroughly wash out any debris that may be left. The spray that I use is echefolta, 1 ounce; dioxygen, 5 ounces, and water, 4 ounces.—J. V. Conzett in *Review*.

**A Varnish for Impressions.** Sodium silicate and ammonium hydroxide equal parts, forms a varnish that is indispensable for plaster molds, impressions, etc., and is inexpensive. For impressions—apply in the same manner as you would shellac. A coloring matter, as carmine, may be added so that the division line may be readily seen. Separate in the same manner as with shellac. When used on models in flask before packing the rubber the plaster readily separates from the plate after vulcanizing, leaving it clean and smooth.—T. A. Leach in *Review*.

**Artificial Platina on Brass.** The green coating—"Patina"—found upon bronze objects, especially such as have lain buried for some time, is not only pleasing to the eye, but also serves a practical purpose, in that the metal beneath is protected from further corrosion.

Brass objects can be coated, as well as bronzes, by means of a solution made according to the following formula:

| | |
|---|---|
| Copper | 30 gms. |
| Nitric acid, conc | 60 gms. |
| Acetic acid, 6 | 600 gms. |
| Ammonium chloride | 11gms. |
| Ammonia water | 20 gms. |

The copper is dissolved in the nitric acid, and, as soon as solution is effected, the other ingredients are added. The solution must be allowed to stand several days before using.

The objects to be coated are either dipped into the solution for a moment, or the solution is applied to the surface by means of a brush. They are then allowed to dry, and are finally covered with a thin coat of linseed oil.

**Would Consolidate Registry Boards.** That the State would annually save thousands of dollars and that the work would be accomplished in just as methodical and accurate a manner as under the present arrangement, was the argument advanced by Senator Allan T. Treadway, of Berkshire, before the committee on public health in support of his bill to abolish the present individual board of registration in medicine, pharmacy, dentistry, veterinary and embalming and the establishment of one registrar appointed by the Governor to do the same work under guidance of the State Board of Health.—*Boston American.*

**Diseases in Public Schools.** Dr. W. C. Noble in the December *Journal* gives the diseases which have necessitated the removal of scholars from the Montclair, N. J., public schools: During the past year, I find that 33 out of a total of 192 exclusions were cases of tonsilitis, 7 cases of measles, 18 cases of true scarlet fever and 19 cases of suspected; 33 cases of contagious eye diseases—trachoma and purulent conjunctivitis—37 cases of vermin and the rest scattering. There were 1,768 male pupils and 1,781 female pupils examined. Of these, 2,788 were reported by the inspectors as negative, 569 referred either to parents, guardians or physicians and 192 excluded.

**Flux for Base Metals.** Rosin is the best flux for lead. Its flashing point is sufficiently high to permit the lead to be thoroughly melted without the rosin taking fire. Should it take fire it is a sign that the metal is too hot. It not only protects the metal from oxidation, but it cleans the metal by dissolving the oxides already formed; it moreover has the merit of being inexpensive. Paraffin, wax, burgundy pitch, gum, dammar, gum copal, etc., have the same properties as rosin. They are no better, and as they are much more expensive, there is nothing to be gained by their use.

Tallow and palm oil, which are so extensively used with tin, are not suitable for lead; their flashing point is so low that they readily take fire; they are, however, ideal fluxes for tin, which has a much lower melting point. Sal ammoniac is the only practical flux for zinc; it acts differently than does rosin, etc., with lead. The sal ammoniac and the oxides of zinc react the one with the other; ammonia gas and water are formed, which passes off; zinc chlorid is also formed, and it is this which does the work. It is only in exceptional cases that these fluxes are needed in the dental workroom; the base metals are so little used, and they are so inexpensive, that it is usually economy to submit to a little waste rather than to bother with fluxes.—The *Brass World*, November, 1907, page 363.—*Brief.*

**Canadian Oral Prophylactic Association.**

The Canadian dentists have perfected an organization and, through a Chemical Manufacturing Association, are placing on the market a tooth powder and mouth-wash which they can recommend to their patients and from the sale of which they will make a small profit, which they will apply to some form of benevolence that is connected with professional interests. Just what this will be, will largely depend on the success of their venture financially. There doesn't seem to be any crying need for such a movement on this side, as we have a great number of very good mouth toilet articles. The only threatening tendencyto do such a thing here, is the efforts being made to use dentists as advertising means through appeals to their mercenary inclinations, by some form of stock interests. Sometimes a corporation becomes too successful and because its infatuation with its own developement makes the mistake of overreaching itself, by going out of its way to encroach on the prerogatives of others, and so bring itself into antagonisms with its creators and friends. The Standard Oil Company was never criticised until it went out of its legitimate field to take up business enterprises that did not legitimatly belong to it. The department stores and mail order commercial houses are fast becoming obnoxious because of their business methods. It is barely possible that our Canadian friends may find that the work they are undertaking can be safely left to the hands of competent druggists and chemical manufacturing concerns.

**The Toxic Effects of Quinosol Cresol and Lysol.**

Th. Weyl has studied the toxic effects of these three substances upon rabbits, and publishes the results of his investigations n the *Vierteljahresschrift F. Gerichtliche Medicin.*

He finds that in the stomach of the rabbit, lysol and cresol are equally poisonous, but when administered hypodermically, quinosol is more poisonous than lysol, while cresol is less poisonous than either of the other two. Injected into the peritoneum, quinosol has a far less toxic effect than lysol or cresol.

Expressed in numbers, the minimum lethal doses were as follows:

Introduced into the stomach cresol is 163 per cent. more poisonous than lysol, and 135 per cent. more poisonous than quinosol.

When hypodermically injected, cresol is found to be 136 per cent. less poisonous than lysol, and 371 per cent ,less poisonous than quinosol.

The results of the inter-peritoneal injections show that cresol has the same toxic value as quinosol, and is 51.5 per cent less poisonous than lysol.

In view of the large number of cases of lysol poisoning that have occurred in Germany within the past year, and the consequent restrictions placed upon its sale by the government, the investigator urges that the sale of quinosol, whose poisonous properties are equal to those of lysol, if not greater, be likewise regulated.—*Pharm. Zentralh.*

**Action of Iron Preparations on the Teeth.** Dr. Morgenstern has made an exhaustive study on the action of iron salts and ferruginous waters on the teeth. He made thin sections of the teeth of man, the ox and the calf, and immersed these in solutions of the salts, 100 cc. of the solution being used in each case. The period of experimentation extended over two to four months, and the sections were examined twice weekly under the microscope. From his results, as given in the *Therapeutische Monnatshefte*, it is apparent that ferratin, ferrum reductum, liquor ferri albuminati, and liquor ferri saccharati have no deleterious effect on the teeth. Ferri citras, Ripoldsauer Stahlquelle and Cudowa-Eugenquelle mineral waters have a faint action on the teeth. A partial decalcification and blackening of the teeth is caused by tincture ferri pomati, the pyrophosphate, lactate and sulphate of iron, and the mineral waters of the Elsterquelle, Pyrmonterquelle and Schwalbacherquelle.

Ferrous iodide and chloride are especially injurious, a ten per cent solution of which will remove the enamel in a few days. He also finds that the absolute per cent of iron in a mineral water does not determine the injurious effect thereof on the teeth. This action is largely subordinated to the presence of salts of the earths, which seem to promote the corrosive action of the iron. The iron exhibited reappears in the saliva, but, as it is now in the form of an albuminate, it has no effect on the teeth. In conclusion he states, as has long been quite generally practiced, liquid iron preparations should always be taken through a glass tube by the patient. Iron preparations are best given in pill form, or powdered in gelatin capsules.—*Dental Era.*

**Secret Formula.** No dentist will admit that it is desirable to take instruction on the manipulation of materials used in dentistry from the manufacturers, but so long as dentists continue to use materials, the composition of which they know nothing about, they must take their instructions from the manufacturer. There is not a cement on the dental market the composition of which is known to the profession. The whole line of gutta percha and stoppings are as a sealed book to the users. Alloys for the making of amalgam are as little known to the users as the constituents of Sanatol.

Not one of the materials used in filling teeth—gold not excepted—but should be manipulated in accordance with its composition. The manipulation of a high grade silver alloy should be different from the manipulation of a low grade alloy. And since the manufacturer is the only person who knows the constituents he is the only one who can give the necessary instruction, but if he should not know that his material should be manipulated differently from one of another composition then the profession must suffer. The only solution is not to use a material the composition of which is not given. Dentists often make a great deal of talk about patent medicines, etc., while they accept gold solders marked 18 karat, which are in reality about 12 karat, and do not know whether the gold is alloyed with silver lead, tin, zinc or copper, or scrap iron. They buy an alloy which is supposed to be made after Black's formula, but mixes like putty, and sets as slowly as cement used in a pavement. If dentists would demand the composition of their materials from the manufacturers there would be fewer leaky amalgam fillings and broken bridges. Cement fillings would last longer and inlays would be less frequently lost. It is high time the profession should demand the composition of the materials they use, and should support those who give the composition. The only compounds used or recommended by the dentists in Canada which they know the constituents of are "Hutax" preparations.—*Dominion Journal.*

**Specialization.** Specialization in the various branches of the healing art is more and more pushed to the foreground. No one individual can ever hope to master all parts of one subject, the field of human endeavor is too extensive and complicated. What is true of general medicine, holds partially good in dentistry. In a recent address before the section of stomatology of the A. M. A., Dr. W. S. Bryant, of New York, made the following very significant remarks upon this important phase of the subject: "The dental orthopedist has only just begun to attract attention and orthopedics of the nose, we might say, has scarcely as yet come into existence; but there are indications that it will outstrip all other branches of corrective surgery because it directly affects the whole organism and the mental capacity instead of the less important locomotor functions.

While it is true that keeping abreast with the rapid advance that dentistry has made within the last few decades has proved to be a difficult matter for every man, it is equally true that too much specialism has its serious drawbacks. To start with, a dental practitioner, possessing only the dental degree, has no right to be classified as a specialist of a branch of medicine. To be a specialist signifies to be "one who has a special knowledge of some particular subject, thus the ophthalmologist, the neurologist, etc., are specialists of medicine" or in other words, to be a medical specialist means to be primarily the possessor of such medical knowledge according to the conception of the law which entitles one to practice medicine in all its branches by virtue of the state medical license. A person holding a state medical license can lawfully practice dentistry, while on the other hand, a person holding a state dentist's license cannot legally practice medicine. To classify, therefore, a D.D.S. as a medical specialist is a *contradictio in adjecto.* Socrates, in the Charmides of Plato, makes this statement: "And this is the reason why the cure of many diseases is unknown to the physicians of Hellas, because they are ignorant of the whole, which ought to be studied also; for the part can never be well unless the whole is well." If we apply this statement to the practice of a specialty in dentistry, it means that no one should specialize unless he possesses a full knowledge of general dentistry, acquired in an actual practice of at least five years duration. At the expiration of this time he is capable of selecting the line of work which is best adapted to his individuality and at the same time ke knows enough general dentistry to keep him from making mistakes as a specialist when dealing with cases that require general as well as special treatment. With all due respect to the specialist, there is probably no doubt that many specialists suffer from a narrowness of view that often adversely operates against the interest of the patients. This is especially true in the beginner who enters into the domain of a specialty directly after graduation. The benefits of a broad conception of a chosen profession can only be gained by mastering its details and, according to the theory of natural selection, proficiency in a part thereof will manifest itself in an early hour.—H. Prinz in *Era.*

**The Evils of Quack Medical Advertisements.** If all the injury done to humanity directly and indirectly by quack medical advertisements were to be answered for by the orthodox fire and brimstone, there would'nt be fuel enough in the universe to supply the demand. More sickness has been developed by reading this trash than has ever been cured by the so-called remedies it advertises, and more money has been squandered by the people in buying the worse than useless concoctions thus exploited than would have paid the national debt many times over or built homes from one end of the land to the other for the aged and helpless. It is a positive evil which has planted its poison in the public mind for so many years and to such an extent that the wonder is that legislation has not long ago been passed to suppress it.

It is to be hoped that ultimately the committee of One Hundred on National Health in conjunction with the Public Health Defense League will be able to accomplish something in this direction. If President Roosevelt's statement is true that, "Our national health is physically our greatest national asset," then it would seem to be imperative that those entrusted with our national welfare should take cognizance of this tangible and perpetual menace to the public health.

In almost every newspaper and magazine in the land are printed advertisements the reading of which are calculated to make many well people sick. The heralding of disease before the public mind at all times in glaring headlines is not conducive to health or happiness. The adroit description of "symptoms" in many of these advertisements is not only misleading but in some instances positively diabolical, and should be rigidly suppressed.

Last summer I was riding on a railway train which was largely occupied by farmer folk and the good substantial middle class. At a station a young man boarded the car and distributed among the passengers a pamphlet describing all sorts of diseases possible—and impossible, with of course the sovereign remedy. There was not a single individual who read that pamphlet, as most of them did, who felt as well after reading as before, and the disturbance of peace of mind and the amount of money squandered on that one train will never be known. But it is safe to say that every individual was made less happy by reading "symptoms," and that enough money was spent to pay for printing the pamphlet, for employing the young man, for keeping up an immense establishment to manufacture the "dope," and for a beautiful profit to the proprietors who run it. Men are not in the patent medicine business for their health, nor for the health of the public. They are there for the dollars they get out of it, and most of them care little more for the welfare of the public than does the hold-up man who attempts to rob the people—in another way.

It is a crying shame on our civilization that an evil of such vast proportions and so far-reaching in its effects should be allowed to menace the public health as this has done for so many years. Quackery of all kinds is bad, but the quackery which continually fosters disease without curing it is a positive crime.—C. N. Johnson in *Review*.

**Inlay Model Making History.** I think we are all interested in the history of casting, and I have here, through the kindness of Dr. Edward C. Kirk, an extract from an old work published in 1846, in which Charles Holtzapffel shows that the idea of casting in a matrix from which the model or pattern has been burned is not a new one.

While we are indebted to Dr. Taggart for bringing the idea of casting to the attention of the dental profession, the idea is one that really belongs to some one far ahead of us.

Dr. Lee (reading extract from Turning and Mechanical Manipulation. By Charles Holtzapffel. Vol. 1. Published 1846):—"Some objects which are either exceedingly complex in their form, or soft and flexible in their substance, and which do not therefore admit of being moulded in sand, in the ordinary manner of figure casting, may be moulded for a *single copy*, provided the originals consist of substances which may be either readily melted or burned into ashes.

"A cavity is made in the sand of the moulding-trough, a little larger and longer than the object, or else a wooden box of appropriate size is procured, in the midst of which the wax model may be placed; to the end of the model is added a piece to represent the runner, which will be required for introducing the metal. The composition of one-third plaster of paris and two-thirds brickdust, mixed with water, the same as for the core of the bust, is then poured in, entirely to surround the model. The mould is first slowly dried, it is then inverted and made warm to allow the wax to run out, after which it is annealed, or burned to redness, and lastly, when cooled, it is buried in sand and filled with metal. The method necessarily throws the chance of success upon a single trial as the model is destroyed.

"Should the face of the casting be required to be particularly smooth, a small quantity of brickdust is washed (in the manner practiced with emery, and to be explained) and mixed with very fine plaster; a coat of this brushed over the model, which excludes air bubbles, the model is quickly placed in its cavity, and the coarser mixture is poured in as before.

This mode was practiced in casting the feather of the equestrian statue of George III., erected in Pall Mall, London. Several beautiful brass castings of vegetable bodies, as ears of wheat, and the thick fleshy plants, such as cockscombs, etc., recently exhibited in the Adelaide Gallery appeared to have been thus produced, or with the previous composition.

"The above method exactly corroborates a mode long since described as being suitable to casting copies of small animals or insects, parts of vegetables and similar objects, these are to be fixed in the center of a small box by means of a few threads attached to any convenient parts, one or two wires being added to make air-holes, and ingates for the metal. A small quantity of river silt or mud, which had been carefully washed, was first thrown in and spread around the object by swinging the box about; and when partly dry, successive but coarser coats were thrown in, so as ultimately to fill up the box. When it had become thoroughly dry, the wires were first removed from the earthy mould, it was then burned to reduce the object to ashes, and, when every particle of the model had been blown out, it was ready to be filled with metal."—A. P. Lee in *Brief*.

